# Genomic adaptation of Ethiopian indigenous cattle to high altitude

**DOI:** 10.3389/fgene.2022.960234

**Published:** 2022-12-09

**Authors:** Endashaw Terefe, Gurja Belay, Jianlin Han, Olivier Hanotte, Abdulfatai Tijjani

**Affiliations:** ^1^ Department of Microbial Cellular and Molecular Biology (MCMB), College of Natural and Computational Science, Addis Ababa University, Addis Ababa, Ethiopia; ^2^ International Livestock Research Institute (ILRI), Addis Ababa, Ethiopia; ^3^ Department of Animal Science, College of Agriculture and Environmental Science, Arsi University, Asella, Ethiopia; ^4^ Livestock Genetics Program, International Livestock Research Institute (ILRI), Nairobi, Kenya; ^5^ CAAS-ILRI Joint Laboratory on Livestock and Forage Genetic Resources, Institute of Animal Science, Chinese Academy of Agricultural Sciences (CAAS), Beijing, China; ^6^ Centre for Tropical Livestock Genetics and Health (CTLGH), The Roslin Institute, The University of Edinburgh, Midlothian, United Kingdom; ^7^ School of Life Sciences, University of Nottingham, Nottingham, United Kingdom

**Keywords:** high altitude, hypoxia, Ethiopian cattle, adaptation, candidate gene, convergent evolution

## Abstract

The mountainous areas of Ethiopia represent one of the most extreme environmental challenges in Africa faced by humans and other inhabitants. Selection for high-altitude adaptation is expected to have imprinted the genomes of livestock living in these areas. Here we assess the genomic signatures of positive selection for high altitude adaptation in three cattle populations from the Ethiopian mountainous areas (Semien, Choke, and Bale mountains) compared to three Ethiopian lowland cattle populations (Afar, Ogaden, and Boran), using whole-genome resequencing and three genome scan approaches for signature of selection (iHS, XP-CLR, and PBS). We identified several candidate selection signature regions and several high-altitude adaptation genes. These include genes such as *ITPR2, MB,* and *ARNT* previously reported in the human population inhabiting the Ethiopian highlands. Furthermore, we present evidence of strong selection and high divergence between Ethiopian high- and low-altitude cattle populations at three new candidate genes (*CLCA2, SLC26A2,* and *CBFA2T3*), putatively linked to high-altitude adaptation in cattle. Our findings provide possible examples of convergent selection between cattle and humans as well as unique African cattle signature to the challenges of living in the Ethiopian mountainous regions.

## Introduction

Ethiopia is endowed with diverse agro-climatic regions and altitudes that range from the lowest Afar depression (−160 m above sea level, masl) to the highest Semien Mountain (4,600 masl). The Ethiopian highlands are commonly found in the central part of the country, on both sides of the Rift Valley, extending from the Semien Mountain in the North to the Bale Mountain in the Southeast. Cold temperatures and humid weather are characteristics of the high-altitude plateaus in Ethiopia. Mixed livestock farming and crop cultivation are major agricultural activities for livelihoods. These environments are characterized by some unique agricultural production activities and food sources, providing cash income to the local communities (Asresie and Zemedu, 2015). Likewise, they have contributed to the diversity of Ethiopian livestock.

Human populations started to occupy the Ethiopian high plateau by migrating from the Rift Valley to the Bale Mountain in the Middle Stone Age around 50–30,000 years ago (Ossendorf et al., 2019). Overtime, people living in such environments have become adapted to high-altitude stressors, including hypobaric hypoxia, ultraviolet light, cold temperature, and oxidative stress (Beall et al., 2002; San et al., 2013; Debevec et al., 2017; Yang et al., 2017; Storz, 2021). The possible genetic basis of human adaptation to high altitudes in Ethiopia has been previously reported (Beall et al., 2002; Alkorta-Aranburu et al., 2012; Scheinfeldt et al., 2012; Huerta-Sánchez et al., 2013; Edea et al., 2019; Wiener et al., 2021). Likewise, evolutionary adaptations of livestock exposed to high altitudes are expected to be associated with major changes in the anatomy and physiological functions following a long period of acclimatization. For example, the yak *Bos grunniens* possesses a larger heart and lungs as compared to cattle (Wang et al., 2016), leading to a high amount of inhaled air to supply sufficient oxygen to the respiratory cell system. Uteroplacental oxygen flow at the fetal stage (Simonson, 2015), higher hemoglobin concentration in blood (Alkorta-Aranburu et al., 2012; Scheinfeldt et al., 2012), pulmonary vasoconstriction (Wang et al., 2016), ability to avoid altitude sickness (Dolt et al., 2007), calcium metabolism (Wang et al., 2015), and better foraging ability and energy metabolism (Qiu et al., 2012; Edea et al., 2014) may all contribute to the high-altitude adaptation in cattle and other livestock species.

High-altitude adaptation in animals relies on their genetic background attained through natural selection. Hypoxia induced factors such as HIF-1a and its paralogs of HIF-2a and HIF-3a are oxygen regulating factors in a hypobaric hypoxia environment and are thus considered candidate genes for high-altitude adaptation. The HIF-1a gene is over-expressed in cattle, yak, humans, and the Tibetan gray wolf living at high altitudes (Newman et al., 2011; Bigham and Lee, 2014; Zhang et al., 2014; Wang et al., 2016; Verma et al., 2018; Werhahn et al., 2018). The HIF-1a pathways include the endothelial PAS domain 1 (*EPAS1*), vascular endothelial growth factor-A (*VEGF-A*), endothelial converting enzyme-1 (*ECE1*), glucose transport members 1 (*GLUT-1*), hexokinase 2 (*HK2*), and nitric oxide synthesis (*NOS2*) genes. These are all expressed in cattle adapted to high altitudes (Verma et al., 2018), and they play an important role in maintaining oxygen homeostasis and glucose metabolism in mammals (Hu et al., 2006; Majmundar et al., 2010). Hypoxia-related genes, including *EPAS1*, *RYR2*, and *ANGPT1*, were identified in high-altitude Tibetan gray wolves, and they were associated with the HIF signaling pathway, ATP binding, and response to oxygen-containing compounds (Zhang et al., 2014). Using the Illumina bovine low-density 50K SNP array, a study on the Ethiopian cattle population living at an altitude of 2,400 masl identified energy metabolism (*ATP2A3*, *CA2*, *MYO18B*, *SIK3*, *INPP4A*, and *IREB2*) and response to hypoxia (*BDNF*, *TFRC*, and *PML*) genes as candidate genes to the adaptation of cattle to the high-altitude environments (Edea et al., 2014).

Physiological and genomic landscape studies have revealed convergent evolution in several species to independently adapt to high altitudes in different geographic locations across the world (Scheinfeldt et al., 2012; Huerta-Sánchez et al., 2013; Azad et al., 2017; Witt and Huerta-Sánchez, 2019; Friedrich and Wiener, 2020). For example, human populations in the Tibetan, Andean, and Ethiopian highlands shared common candidates selected regions and genes linked to high-altitude adaptation, such as *PPARA* and *EDNRA* (Scheinfeldt et al., 2012; Simonson, 2015; Witt and Huerta-Sánchez, 2019). However, *ARNT2* and *THRB* were uniquely identified in the Ethiopian population (Scheinfeldt et al., 2012).

Only a few studies have reported the environmental adaptations of Ethiopian cattle at the full autosomal genome level (Kim et al., 2017; Kim et al., 2020). Though the bovine low-density SNP array (Edea et al., 2014) was the first to investigate Ethiopian cattle adaptation to different environments including hypoxia. However, it did not include high altitude adaptation of cattle population living at an altitude of >3,000 masl. Therefore, this study aimed to identify signatures of positive selection for high altitude adaptation in Ethiopian cattle using whole-genome resequencing. For this purpose, we selected three cattle populations from the highest mountainous areas (>3,000 masl) of the country (Bale, Choke, and Semien Mountain areas) (Table 1), and three Ethiopian cattle populations living at low altitudes.

**TABLE 1:** Sampling location and cattle population description in the high and low altitudes.

Category	Breed	Region (district)	Location	Altitude (masl)	GPS		Climate
					Latitude (degree)	Longitude (degree)	
High altitude	Bale	Oromia (Bale)	Bale Mountain	3,586	6.77	39.75	Cold humid, highland
	Choke	Amhara (East Gojam)	Choke Mountain	3,410	10.60	37.84	Cold humid, highland
	Semien	Amhara (North Goder)	Semien Mountain	3,732	13.23	38.13	Cold humid, highland
Low altitude	Afar	Afar	Melka Were/Asayta	729	9.34	40.17	Hot and dry lowland
	Boran	Oromia (Borena)	Dubulk	1,368	4.55	38.10	Hot and dry lowland
	Ogaden	Ogaden	Jigjiga	1,200	09.58	41.85	Hot and dry lowland

## Materials and methods

### Cattle populations and whole-genome resequencing

**FIGURE 1:**
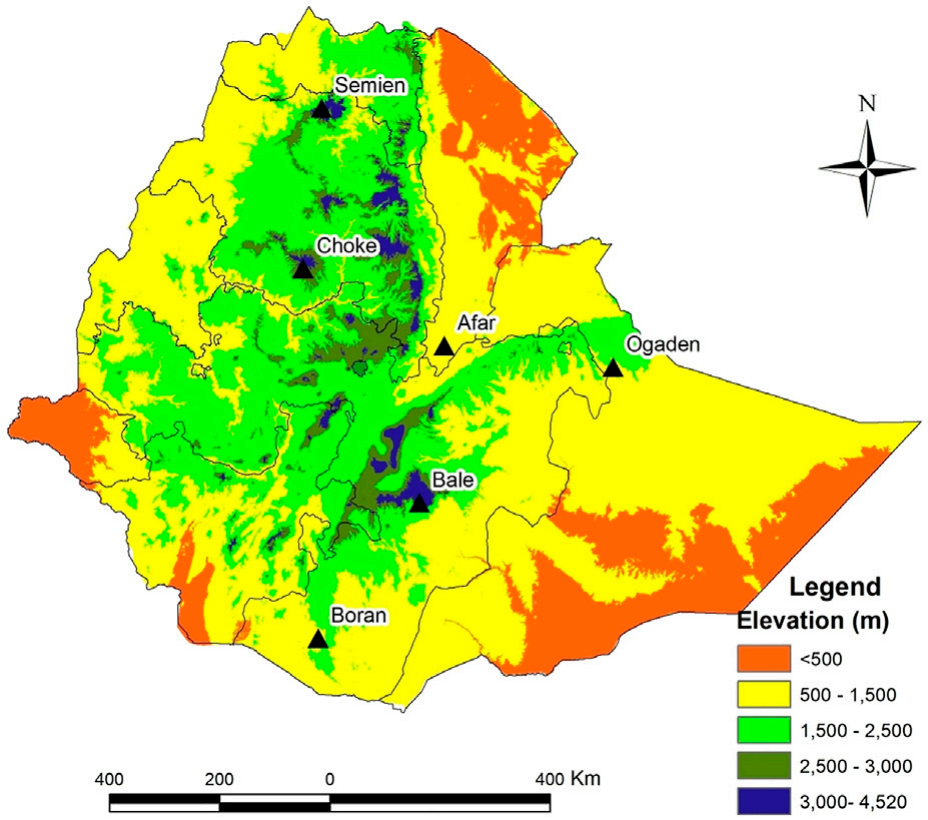
The elevation map of Ethiopia and sampling locations for high and low-altitude cattle populations.

The study involved a comparative analysis of indigenous cattle distributed at high altitudes (>3,000 masl) and low altitudes (<1,500 masl) in Ethiopia (Table 1). The high-altitude populations included Bale (*n* = 10) sampled in the Bale district (Bale Mountain, ∼3,586 masl), Semien (*n* = 10) in the Gondar district (Semien Mountain, ∼3,732 masl), and Choke (*n* = 10) in the Gojam district (Choke Mountain, ∼3,410 masl). The low-land populations included Afar (*n* = 11) sampled in the Afar district (∼729 masl), Ethiopian Boran (*n* = 10) from the Borana district (∼1,368 masl), and Ogaden (*n* = 9) from the Ogaden district (∼1,200 masl) (Figure 1).

Whole blood samples were collected aseptically from the jugular veins of unrelated individuals into 10 ml vacuum tubes containing EDTA. Genomic DNA was extracted using the QIAGEN DNeasy Blood and Tissue Kit (https://www.qiagen.com/us/) following the manufacturer’s protocol. The DNA integrity was checked by a 1% agarose gel electrophoresis and observed under a UV light-based gel viewer. The concentration and quality of DNA for each sample were checked by using a DeNovix DS-11 FX Series Spectrophotometer/Fluorometer (DeNovix Inc., Wilmington, DE, United States). DNA samples (>50 μg/μl) were then shipped to Novogene, China (https://en.novogene.com/services/research-services/genome-sequencing/whole-genome-sequencing/animal-plant-whole-genome-sequencing-wgs/), where whole-genome sequencing was performed on an Illumina NovaSeq 6000 Platform (Illumina, San Diego, CA, United States) to generate 150 bp of paired-end reads. We included Gir (GenBank accession no. PRJNA343262), Angus (PRJNA318087), Muturu (PRJNA386202), and Butana (PRJNA574857) cattle for comparative analyses, following the same sequence quality control and variant calling procedures.

### Read mapping and variant calling

The quality control of raw sequencing reads was performed using the *FastQC v0.11.9* program (https://github.com/s-andrews/FastQC/releases/tag/v0.11.9). Qualified raw reads were processed for initial trimming and filtering of the low-quality reads by removing adapters, short reads (sequence length <35 bp), and reads with low sequence base quality (quality score <20) using the *Trimmomatic v0.38* tool (Bolger et al., 2014). Clean reads were mapped to the latest taurine cattle reference genome of ARS-UCD1.2 (Shamimuzzaman et al., 2020) using the *BWA-MEM* algorithm of Burrows-Wheeler Aligner (*bwa v0.7.17*) (Li and Durbin, 2010).

Mapped reads were sorted and indexed using the *samtools v1.8* (Li et al., 2009) to produce a BAM file. Alignment sorting by coordinate and marking of potential PCR and optical duplicates were carried out using the *MarkDuplicates* tool in *Picard v2.18.2* package (http://picard.sourceforge.net). Base quality score recalibration (BQSR) and haplotype caller analysis were performed using the *GATK v3.8-1-0-gf15c1c3ef* according to its best practice workflows (McKenna et al., 2010). The known variants of ARS1.2PlusY_BQSR_v3.vcf.gz provided by the 1,000 Bull Genomes project were used for masking known sites for all cattle samples. The *GATK PrintReads* was run to adjust the base quality scores in the data based on information from the table and to produce a recalibrated bam file.

Then, the genomic variant call format (*gVCF*) file for each sample was created using the *GATK HaplotypeCaller* command from the recalibrated bam file, and all samples were combined to obtain a joint genotype file using the *GATK CombineGVCFs*. Finally, the variants were processed for variant recalibration with a 99.9 truth sensitivity filter level using the *GATK* to reduce false discovery rates that minimize the noise created by low standard variants. After all quality checking, approximately 36.6 million biallelic autosomal SNPs were identified and used for downstream analyses.

### Population genetic structure

Principal component analysis (PCA) and admixture modeling were performed, based on the SNP genotypes, to determine the population genetic structure of indigenous Ethiopian cattle living in high and low altitudes. We used Angus, Gir, Muturu, and Butana cattle as reference breeds (European taurine, Asian zebu, African taurine, and non-Ethiopian zebu cattle). The dataset in the vcf file was first converted to a plink format (map, ped, and fam) after pruning the SNPs in linkage disequilibrium (LD) (r^2^ ≥ 0.5), minor allele frequency (MAF) (< 0.05), and missing genotype (call rate > 10%) based on a step-wise procedure using 50 SNPs windows and 10 SNPs steps. After the stringent variant quality check, 5.1 million autosomal SNPs with an average of 98.1% genotyping rate were used for admixture modeling with the *Admixture v1.3.0* software (Alexander et al., 2009) to estimate the ancestry proportion of individual samples. The ancestral proportions in the hierarchical clustering of individual samples were optimized at the *K* values ranging from 2 to 10 and the admixture plot was visualized using the R package. For PCA, we removed SNPs with MAF < 0.01, SNPs with missing genotypes > 10%, and SNP calling rate < 90%. After this filtering, 25.5 million SNPs were used for PCA. The eigenvectors of each sample were calculated using *PLINK 1.9* (Purcell et al., 2007) and the result was plotted with the ggplot2 in the R package.

### Integrated haplotype homozygosity analysis

The phasing and imputation of missed genotypes were estimated per chromosome for each population using the *Beagle v5.1* software (Browning and Browning, 2007). The length of homozygous haplotypes along a chromosome was used to estimate the LD decay. The extended haplotype homozygosity (EHH) from each SNP, which is the probability that two randomly chosen homologous chromosomes carrying the core haplotype of interest are identical by descent (Sabeti et al., 2002), was then calculated. The integrated haplotype score (iHS) compares the integrated EHH between the ancestral allele relative to the derived allele in a population (Voight et al., 2006). It detects selective sweeps when alleles are near fixation. The iHS analysis was done using the *REHH v2.0* R package (Gautier et al., 2017) for alleles with MAF within a population > 0.05. A genomic window of 100 kb and a step size of 50 kb were used to identify candidate regions of selection signatures.

### Cross-population composite likelihood ratio (XP-CLR)

XP-CLR test was done between cattle living at high and low altitudes using the haplotype phased data of each chromosomal window of 100 kb and a step size of 50 kb. The test is based on local allele frequency changes in a genomic region between the two groups. The method is most sensitive to recent selection and detects departures from neutrality that could be compatible with hard or soft selection sweeps (Chen et al., 2010).

### Population branch statistic (PBS)

PBS analysis was employed to detect genomic regions under selection with highly divergent haplotypes (Yi et al., 2010). We run population differentiation (*F*_ST_) analysis using the *vcftools v0.1.15* (Danecek et al., 2011) between the Semien population for the highest altitude representative compared to Afar, Boran, and Ogaden cattle for the low altitude one, and the Sudanese Butana cattle as an outgroup. The top 0.5% of the candidate regions detected by the iHS and XP-CLR tests were used for PBS analysis. We estimated divergence time using a log-transformation of one minus the *F*_ST_ value for each comparison and calculated the PBS value using the method described in Huerta-Sánchez et al. (2013).

### Functional annotation and haplotype structure of candidate genes

The candidate genomic regions were annotated using the taurine cattle reference genome ARS-UCD1.2 (Shamimuzzaman et al., 2020) in the Ensembl database (http://www.ensembl.org/index.html). The Database for Annotation, Visualization, and Integrated Discovery (DAVID, v6.8, https://david.ncifcrf.gov/home.jsp) was used to understand the biological functions and molecular pathways of the candidate genes (Huang et al., 2009), according to the minimum similarity thresholds for enrichment scores at 1.0 and *p* values ≤ 0.05. Further functional annotations were done from the literature published for humans and other vertebrates. Additional structural and functional analyses of the genomic regions of the candidate genes were evaluated using haplotype structure, LD, *F*_ST,_ nucleotide diversity, and STRING protein-protein interaction network database. The haplotype structure was estimated based on pairwise LD heatmap of the SNPs using the *LDBlockShow* (Dong et al., 2020). The *F*_ST_ and nucleotide diversity of the candidate gene regions were estimated using the *vcftools v 0.1.15* (Danecek et al., 2011) in a 10 kb window and 5 kb step size to determine the strength and pattern of selection signatures between the high- and low-altitude cattle. Protein-protein interaction analyses were done using the STRING online platform for the cattle genome reference database (https://string-db.org/).

## Results

### Population genetic structure

**FIGURE 2:**
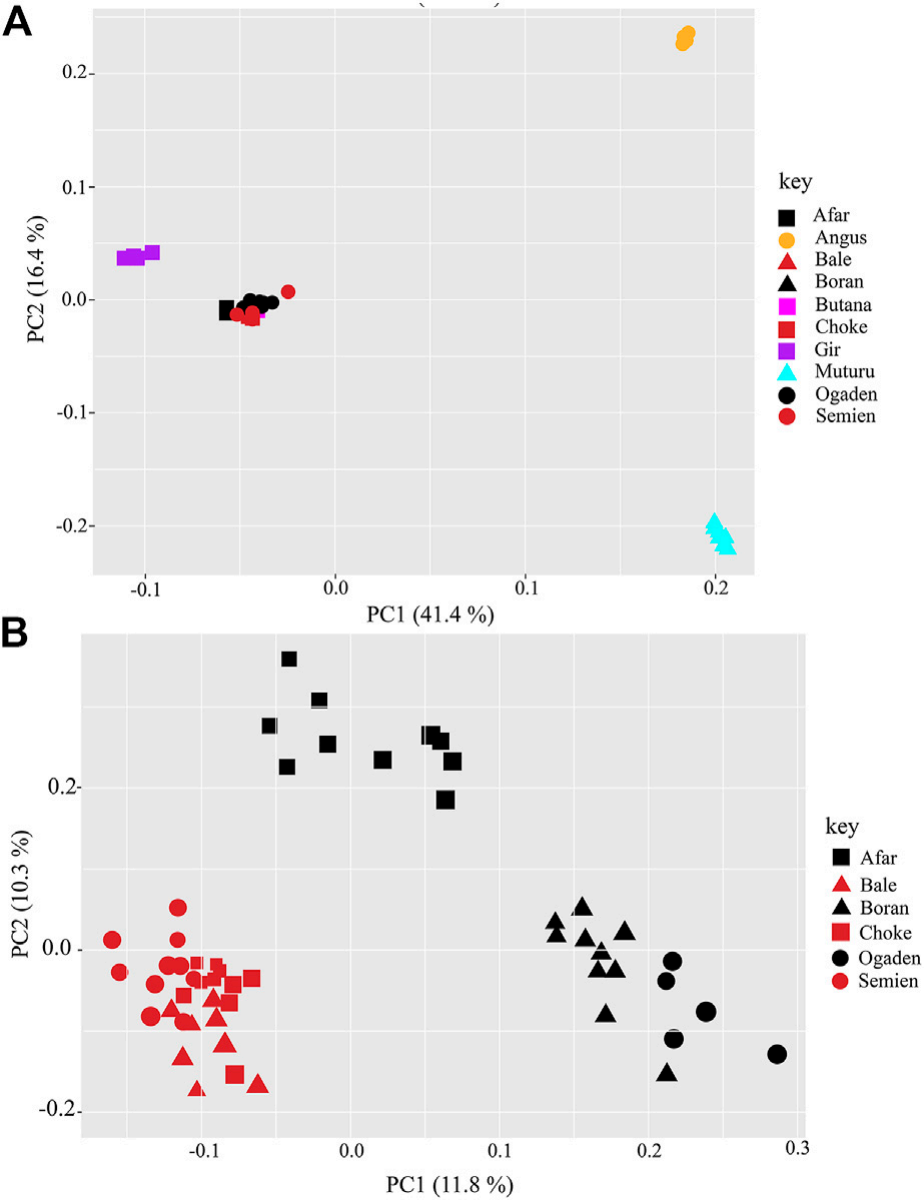
A plot of the first and second principal components (PC1 and PC2) of **(A)** the whole population dataset and **(B)** Ethiopian high altitude (brown color: Bale, Choke, and Semien) and low altitude (black color: Afar, Boran, and Ogaden) cattle populations.

**FIGURE 3:**
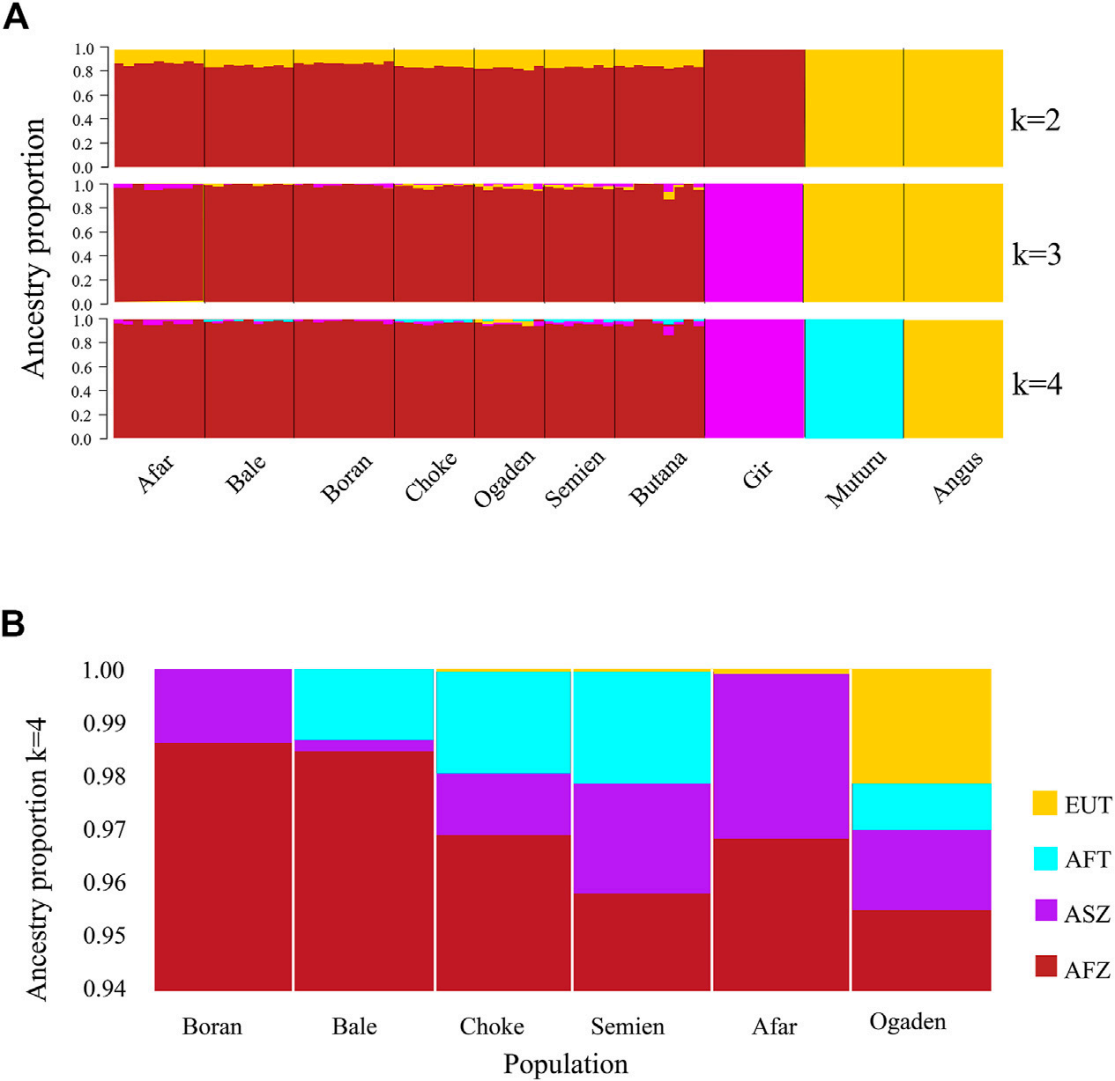
The plot of population genetic admixture analysis. **(A)** Cattle population in the whole dataset and **(B)** Ethiopian cattle ancestry proportion.

The PCA and admixture plots describe the population genetic structure of the indigenous Ethiopian cattle living in the high and low altitudes compared with European taurine cattle (Angus), African taurine (Muturu), Asian zebu cattle (Gir), and African zebu (Butana) (Figure 2A). The first and second principal components (PC) represent 57.8% of the total variation. The first PC (PC1, 41.4%) separates the zebu (Gir, Butana, and Ethiopian cattle) from the taurine (Angus and Muturu), while the second PC (PC2, 16.4%) differentiate the African zebu (Ethiopian cattle Butana) and Muturu from all non-African cattle (Gir and Angus) (Figure 2A). Next, a second PCA was conducted, excluding the reference cattle. The PC1 (11.8%) and PC2 (10.3%) differentiate the Ethiopian high-altitude (HA) from low-altitude (LA) cattle (Figure 2B). The population genetic admixture plot supports the PCA result, which separated cattle populations in the whole dataset into four ancestry clusters (Figure 3A). At K = 4, the genetic ancestry of the Ethiopian cattle was inferred to be 97.0% of African zebu, 1.6% Asian zebu (represented here by the Gir), 1.0% African taurine (Muturu), and 0.4% European taurine (Angus) (Figure 3B). A small shared European taurine component is observed in Bale, Choke, Semien, and Boran cattle. It is, however, higher (2.2%) in Ogaden cattle. The Afar and Boran populations have very little African taurine ancestry proportion. Butana cattle share a similar genetic background to other Ethiopian cattle. To explore potential genome-wide selection signatures for high-altitude adaptation, we analyzed the HA and LA cattle populations separately, following the Ethiopian cattle PCA results (Figure 2B).

### Selection signatures within Ethiopian high- and low-altitude cattle populations

**FIGURE 4:**
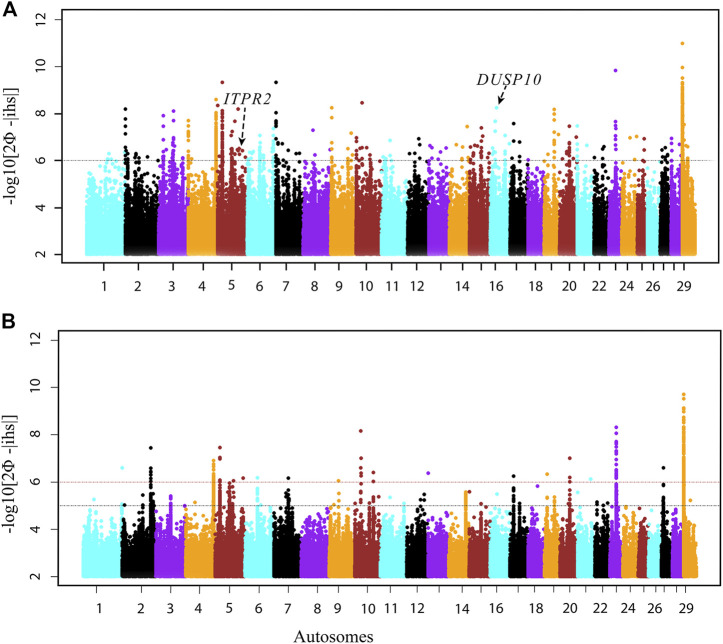
Manhattan plots of genome-wide scans based on the iHS test. **(A)** HA, high-altitude and **(B)** LA, low-altitude Ethiopian cattle populations.

We performed a genomic scan combining the three HA, as well as combining the three LA populations, using the within-population iHS test to identify recent and/or ongoing footprints of natural selection (Vatsiou et al., 2016). Using the *REHH v2.0* R package, we calculated genome-wide iHS for each focal SNP from the phased data (Gautier et al., 2017). Subsequently, we summarized the selection statistics across a sliding 100 kb genomic window with a 50 kb step size. From the empirical distribution of iHS statistics, we applied a *p*-value threshold < 1.0E-6, equivalent to -log10 iHS ≥ 6, to select the candidate regions under selection (Figure 4A, Supplementary Table S1).

There are 144 candidate selected regions across 29 autosomes within the Ethiopian zebu populations living in high altitudes (Supplementary Table S1). These regions vary in size from 150 to 750 kb. They overlap with 264 protein-coding genes based on the Ensembl taurine cattle assembly (ARS-UCD1.2) (Supplementary Table S1). Of these, 28 protein-coding genes were identified within the top 10 *iHS* regions. Most of these genes remain uncharacterized with the exception of *ITPR2* on BTA5 (5:83.45–83.60 Mb, iHS −log10 *p* = 8.19), *DUSP10* on BTA16 (16:25.05–25.20 Mb, iHS −log10 *p* = 8.24), and GTPase IMAP family members 4–7 genes (*GIMAP4*–*7*) on BTA4 (4:112.95–113.35 Mb, *iHS* −log10 *p* = 8.60). These annotated genes are possibly involved in high-altitude adaptation, especially the former two genes with functions linked to the response to hypoxia (*ITPR2*) and oxygen-containing compounds (*DUSP10*), respectively. *GIMAP4*, *GIMAP5*, and *GIMAP7* functions are related to the immune response and hematopoiesis (Chen et al., 2011; Schwefel et al., 2013). They play a significant role in modulating immune functions by controlling cell death and the activation of T cells (Ho and Tsai, 2017).

To contrast the selection signatures of Ethiopian cattle living in high altitudes with the ones living at low altitudes, we performed an additional genomic scan based on the iHS in the three LA cattle populations at the same threshold (*p*-value < 1.0E-6). We identified only 20 candidate regions under selection (Figure 4B, Supplementary Table S2). These regions vary in size from 150 kb to 650 kb. They overlap with 50 protein-coding genes. Twenty-three (∼45%) were common with those identified in the HA populations. The common genes include 14 genes, mostly uncharacterized, present in the top 10 iHS regions, except for the three GIMAP family members, GIMAP 4, 5, and 7 genes. Among the remaining nine common genes, *VEGFC* and *EP300* are possibly linked to the adaptation to high altitudes due to their functions in the vascular system (Herbert and Stainier, 2012; Huerta-Sánchez et al., 2013; Bigham and Lee, 2014; Azad et al., 2017; Zheng et al., 2017). However, due to the fewer candidate regions identified in the LA populations, we decided to increase the iHS threshold to *p* value < 1.0E-7, equivalent to -log10 iHS ≥ 5, which added 113 protein-coding genes, of which 63 were common in both HA and LA populations (Figure 4B, Supplementary Table S2).

### Comparative genomic signatures between Ethiopian high- and low-altitude cattle populations

**FIGURE 5:**
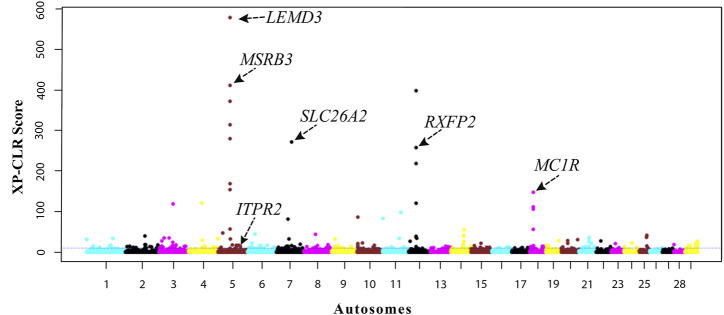
Manhattan plot of genome-wide XP-CLR scores by contrasting the high- and low-altitude cattle populations.

We further investigate the genomic footprints of natural selection in indigenous Ethiopian cattle by contrasting the allele frequency profiles between the HA and LA populations using the XP-CLR test. The top 0.5% XP-CLR scores (XP-CLR > 10) include 216 candidate windows of 100 kb size regions, from which 251 protein-coding genes were annotated (Supplementary Table S3). Unlike many uncharacterized genes within the iHS regions, the top 10 signals identified by the XP-CLR test include seven annotated genes (*MSRB3, LEMD3,* and *WIF1* on BTA5, *SLC26A2, HMGXB3,* and *CSF1R* on BTA7, and *RXFP2* on BTA12) (Figure 5). These are not found within the top high altitude iHS signals. Some have functions that may be related to high-altitude adaptation. For instance, *MSRB3* is involved in cold tolerance in Arabidopsis and high-altitude adaptation in Tibetan dogs and sheep (Kwon et al., 2007; Vaysse et al., 2011; Wei et al., 2016; Witt and Huerta-Sánchez, 2019). This gene was also reported to protect cells from oxidative stress caused by hypoxia (Hansel et al., 2005) as well as from cold and heat stress (Lim et al., 2012). *RXFP2* was reported to control horn type, development, and morphology (Pan et al., 2018; Ahbara et al., 2019; Liu et al., 2020) and linked to high-altitude adaptation to hypoxia in sheep (Guo et al., 2021).

There are 14 genes in common to both XP-CLR and iHS (HA) tests, (*CLCA4*, *CLCA1*, *CLCA2*, *ITPR2*, *ABCB10*, *NUP133*, *GDA*, *GALNT13*, *ENSBTAG00000050002*, *COL14A1*, *AXDND1*, *B3GLCT*, *SOAT1*, and *ENSBTAG00000034225*) (Table 2; Figure 7). These genes could be regarded as promising candidates subjected to natural selection for high-altitude adaptation in Ethiopian HA cattle. On the other hand, no shared candidate gene was found between the LA iHS cattle and the XP-CLR test.

**TABLE 2:** List of overlapping regions and candidate genes identified using the iHS and XPCLR selection scan methods including, gene functions in reported species.

BAT	Region start	Region end	XPCLR score	iHS value	Gene name	Gene function/phenotype	Species	References
2	42.25	42.45	10.99	6.28	GALNT13			
3	17.55	17.6	11.54	6.15	ENSBTAG0	Novel gene/uncharacterized protein	—	—
					0000050002			
3	57.5	57.6	16.01	6.75	CLCA4	Rennin secretion, ion channel activity		Palubiski et al. (2020); Weir and Olschewski, (2006)
					CLCA1	“		“
3	57.55	57.65	12.65	6.75	CLCA2	“		“
5	83.45	83.55	16.65	8.19	ITPR2	Response to hypoxia	Human	Huerta-Sánchez et al. (2013); Jurkovicova et al. (2008); Manalo et al. (2005); Qu et al. (2015)
8	48.4	48.5	43.83	6.23	GDA			
12	29.6	29.7	11.17	6.31	B3GLCT	Carbohydrate metabolic process, protein glycosylation, horn development, environment adaptation	Cattle, sheep	Ahbara et al. (2019); Flori et al. (2019); Pan et al. (2018)
14	81.45	81.55	13.63	6.04	COL14A1	Protein binding, angiogenesis	Mice, rats, sheep, human	Chai et al. (2004); Copple et al. (2011); Wiener et al. (2021); Zhang et al. (2018)
16	60.65	60.75	12.94	7.05	SOAT1	Cholesterol metabolic process	Insects, mice	Guan et al. (2020); Miron and Tirosh, (2019); Zuniga-Hertz and Patel, (2019)
					AXDND1	Response to bone fracture/bone synthesis	human	Pettersson-Kymmer et al. (2013)
28	14.5	15.5	18.81	6.94	NUP133	Regulate mitochondrial function and oxidative stress response	Mouse	Sunny et al. (2020)
					ABCB10	ATPase-coupled transmembrane transporter activity; regulates heme synthesis; Iron metabolism; reactive oxygen species	Mouse, zebrafish, human cell culture	Bayeva et al. (2013); Liesa et al. (2012); Seguin and Ward, (2018); Valverde et al. (2015); Yamamoto et al. (2014)
					ENSBTAG0	Basal transcription, coactivators, and promotor recognition.		
					0000034225			

### Functional annotation of genes under selection in Ethiopian high-altitude cattle populations

We conducted a functional annotation using the DAVID visualization tools, based on the Ensembl taurine cattle assembly (ARS-UCD1.2, to identify GO terms and KEGG pathways for the candidate genes that we detected in the Ethiopian HA cattle populations following iHS and XP-CLR analyses. Genes with fold enrichment >1.2 and *p*-value ≤ 0.05 were considered to be significant (Table 3). Several top candidate genes related to environmental stress such as hypobaric hypoxia, temperature, and UV radiation were clustered into important GO terms, including response to hypoxia (GO:0001666; *p*-value: 9.3E-06), response to oxygen-containing compound (GO:1901700; *p*-value: 1.0E-04), ion channel activity (GO: 0005216; *p*-value: 4.8E-06), glucose homeostasis (GO:0042593; *p*-value: 5.1E-03), and ATPase activity (GO:0016887; *p*-value: 1.3E-03), which are biological processes potentially relevant to high altitude adaptation. These findings are in line with previous reports on cattle and other species adapted to high altitudes (Remillard and Yuan, 2006; Edwards et al., 2007; Shimoda and Polak, 2011; Ge et al., 2013; Veith et al., 2016; Moore, 2017; Hu et al., 2019; Friedrich and Wiener, 2020).

**TABLE 3:** Gene ontology (GO) clustering and enrichment analyses of candidate genes identified by genome-wide iHS and XP-CLR scans in the high-altitude cattle populations.

GO term	Count	*p* value	Fold change	Gene
GO: 0005216∼ion channel activity	15	4.8E-06	4.5	*ITPR2, KCNJ8, GPR89A, TRPM3, CLCA1, NOX5, GRIK3, CACNG2, SLC26A, CLCA4, GABRB2, ABCC9, KCNQ3, CLCA2, TPC3*
GO: 0001666∼response to hypoxia	7	9.3E-04	6.1	*ITPR2, MB, CBFA2T3, SRF, VEGFC, HMOX1, ARNT*
GO: 0034101∼erythrocyte homeostasis	6	1.0E-03	7.7	*MAFB, MB, FOXO3, SRF, HMOX1, ARID4A*
bta04924: Renin secretion	6	2.9E-04	9.9	*ITPR2, CLCA1, PRKACB, CLCA4, CLCA2, GUCY1A2*
GO: 1901700∼response to oxygen-containing compound	19	1.0E-04	2.8	*DUSP10, ITPR2, KCNJ8, IMPACT, NDUFS4, SESN3, AVPR1A, STAT1, PDX1, PTK7, SLC11A1, ADH5, EFNA5, SSTR2, NOX4, HRH4, CRY2, ENPP1, MZB1*
GO: 0008217∼regulation of blood pressure	6	2.1E-03	6.6	*AVPR1A, POMC, GRIP2, ERAP1, ADH5, MYH6*
GO: 0020037∼heme binding	5	2.1E-02	4.7	*MB, HMOX1, CYP4F2, GUCY1A2, ENSBTAG00000048257*
GO: 0016887∼ATPase activity	8	1.3E-02	3.1	*ABCB10, ATP6V0A1, YTHDC2, MYO10, ABCC9, ATP6V1G1, MYH7, MYH6*
GO: 0042593∼glucose homeostasis	6	5.1E-03	5.3	*SESN3, POMC, PDX1, FOXO3, CRY2, NOX4*

Low atmospheric oxygen concentration in the inhaled air causes low oxygen levels in the arterial blood reducing cellular energy production, which leads to cellular stress and then induces several factors to increase oxygen availability to cell mitochondria for energy homeostasis. Physiological homeostasis is established through increasing tissue oxygen supply by mounting vascular smooth muscle tone to withstand fast blood flow pressure by inducing the formation of additional blood vessels (angiogenesis), increasing the number of erythrocytes, and improving heme-binding affinity. Supporting these adaptive mechanisms, we identified candidate genes in the biological processes of erythrocyte homeostasis (GO:0034101; *p*-value: 1.0E-03), heme-binding (GO:0020037; *p*-value: 2.1E-02), and the regulation of blood pressure (GO:0008217; *p*-value: 2.1E-03) enhancement. The increases in erythrocyte, hemoglobin concentration, and heme-binding affinity enable more oxygen transportation to tissues in hypoxia-adapted animals (Storz, 2007; Zhang et al., 2007; Storz and Moriyama, 2008; Storz et al., 2010; Yalcin and Cabrales, 2012; Storz, 2016).

### Identification of candidate genes associated with high-altitude adaptation

**FIGURE 6:**
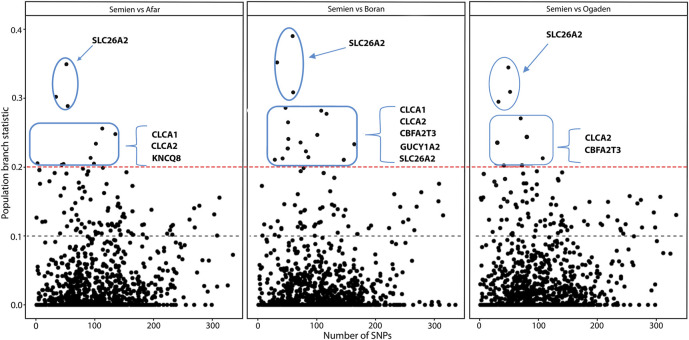
The distribution of the population branch statistic (PBS) values in 10 kb genomic regions as a function of the number of SNPs.

We further analyzed the candidate genes clustered into biological processes relevant to high altitude adaptation (Table 3) using the population branch statistics (PBS). We compared Semien cattle from the highest Ethiopian mountain area to each of the LA cattle populations (Afar, Boran, and Ogaden) using Butana cattle from the Sudanese arid region as an outgroup (see *Materials and methods*). The 10 kb window outliers from the PBS analysis represent the most differentiated genomic regions (PBS value ≥ 0.2). They overlap with *SLC26A2, CLCA1, CLCA2, KCNJ8, GUCY1A2,* and *CBFA2T3* (Figure 6, Supplementary Table S4). These genes have possible roles in ion channel activity, renin secretion, response to hypoxia, response to oxygen-containing compounds, and heme-binding (Table 3). The genomic region within the *SLC26A2* gene was the most differentiated in the PBS scans (Figure 6). *SLC26A2* is a ubiquitously expressed SO4^2−^ transporter with high expression levels in cartilage and several epithelia (Ohana et al., 2012; Park et al., 2014). This gene is involved in body size and male fertility in humans (Kujala et al., 2007; Touré, 2019), and its mutations have been implicated in dwarfism (Yang and Liang, 2021) and dysplasia (Pineda et al., 2013; Zheng et al., 2019; Heidari et al., 2021).

**FIGURE 7:**
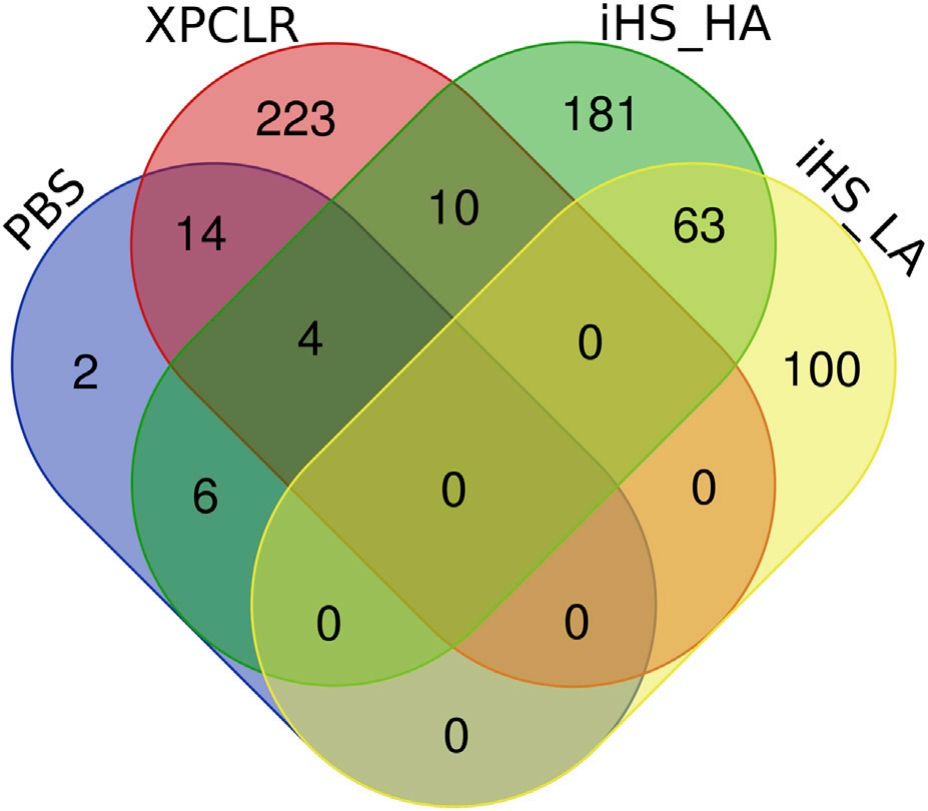
Candidate genes supported by the iHS, XP-CLR, and PBS analyse in high-altitude (HA) and low-altitude (LA) cattle populations.

**FIGURE 8:**
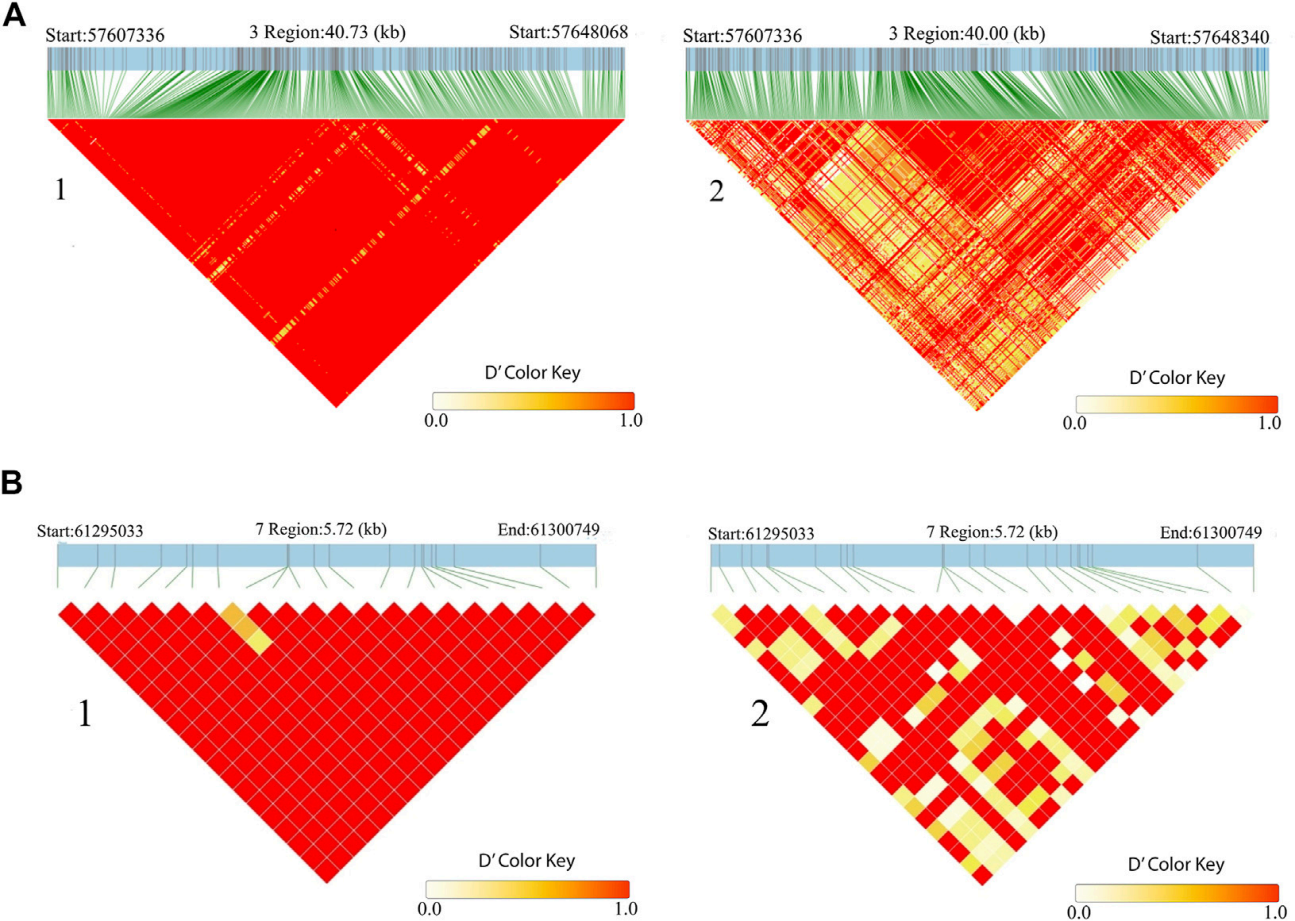
LD block heatmap of the candidate genes of *CLCA2* **(A)** and *SLC26A2* **(B)** in the high-altitude (1) and low-altitude (2) cattle populations.

**FIGURE 9:**
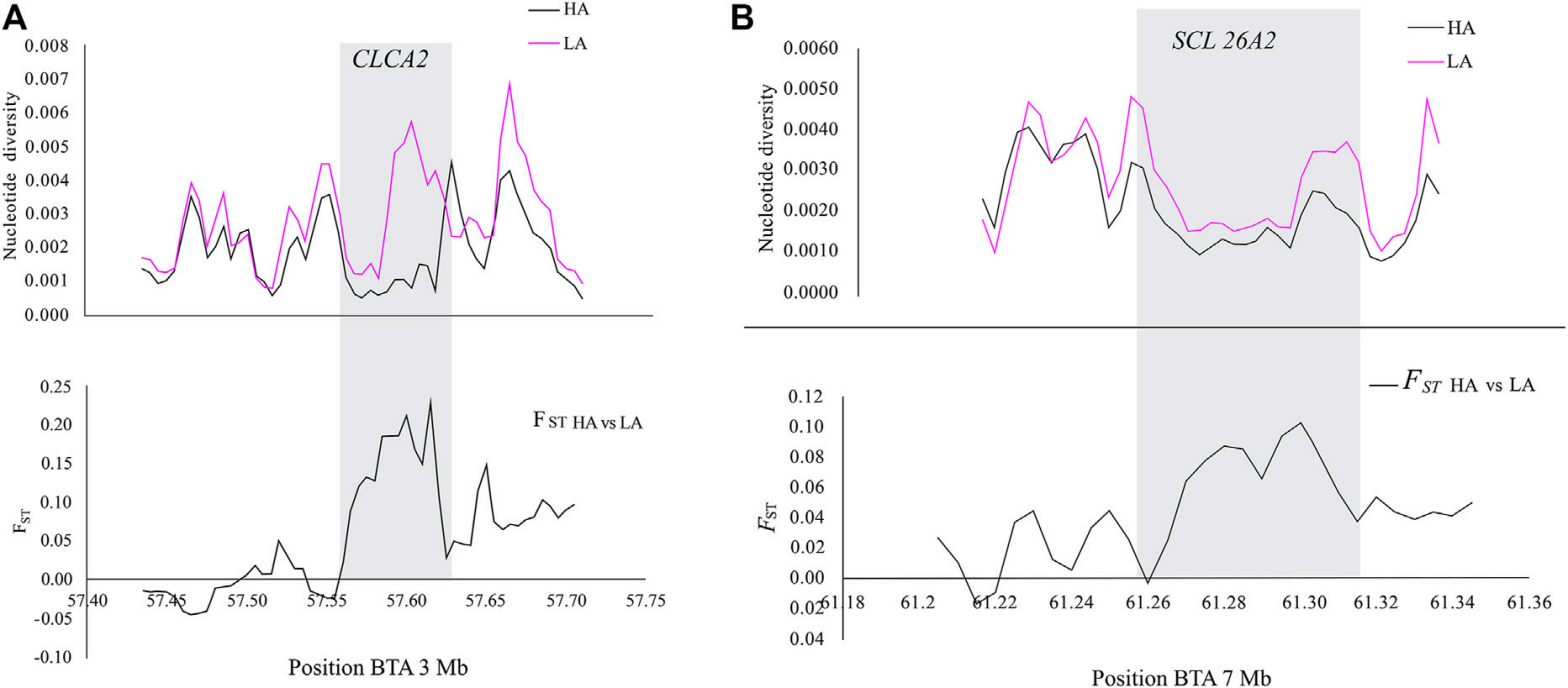
Plots of the nucleotide diversity within and *F*_ST_ values between the genomic regions of *CLCA2* **(A)** and *SCL26A2* **(B)** in high-altitude (HA) and low-altitude (LA) cattle populations.

In addition, *CLCA2* and two other paralogs, *CLCA1* and *CLCA4*, and *ITPR2* were the only four candidate genes detected by the three genomic scans (Figure 7, Supplementary Table S5). Moreover, the variants within the *CLCA2* in the HA populations showed a higher level of linkage disequilibrium (LD) compared to the LA populations (Figure 8). Similarly, the nucleotide diversity and population differentiation plot show the *CLCA2* gene region with significant variation compared to regions of the two paralog genes (Figure 9). Therefore, we considered *CLCA2, ITPR2*, *SCL26A2,* and *CBFA2T3* as strong candidate genes putatively linked to high-altitude adaptation in Ethiopian cattle.

Other genes of interest include GO terms linked to the response to hypoxia (Table 3). These include *MB* (BTA5: 73.81–73.82 Mb), *CBFA2T3* (BTA18: 14.05–14.1 Mb), and *SRF* (BTA23: 16.77–16.78 Mb) from XP-CLR scans results, *ARNT* (BTA3: 19.8–19.9 Mb) and *VEGFC* (BTA27: 8.0–8.1 Mb) from iHS scans results, and *ITPR2* from both XP-CLR and iHS scan results. *ARNT* is involved in the positive regulation of vascular endothelial growth factor (*VEGF*) activation. *VEGFC*, a VEGF homolog, is involved in regulating endothelial cell proliferation and angiogenesis in response to the low oxygen concentration in the arterial blood (Kumar et al., 2011; Herbert and Stainier, 2012; Ramakrishnan et al., 2014).

**FIGURE 10:**
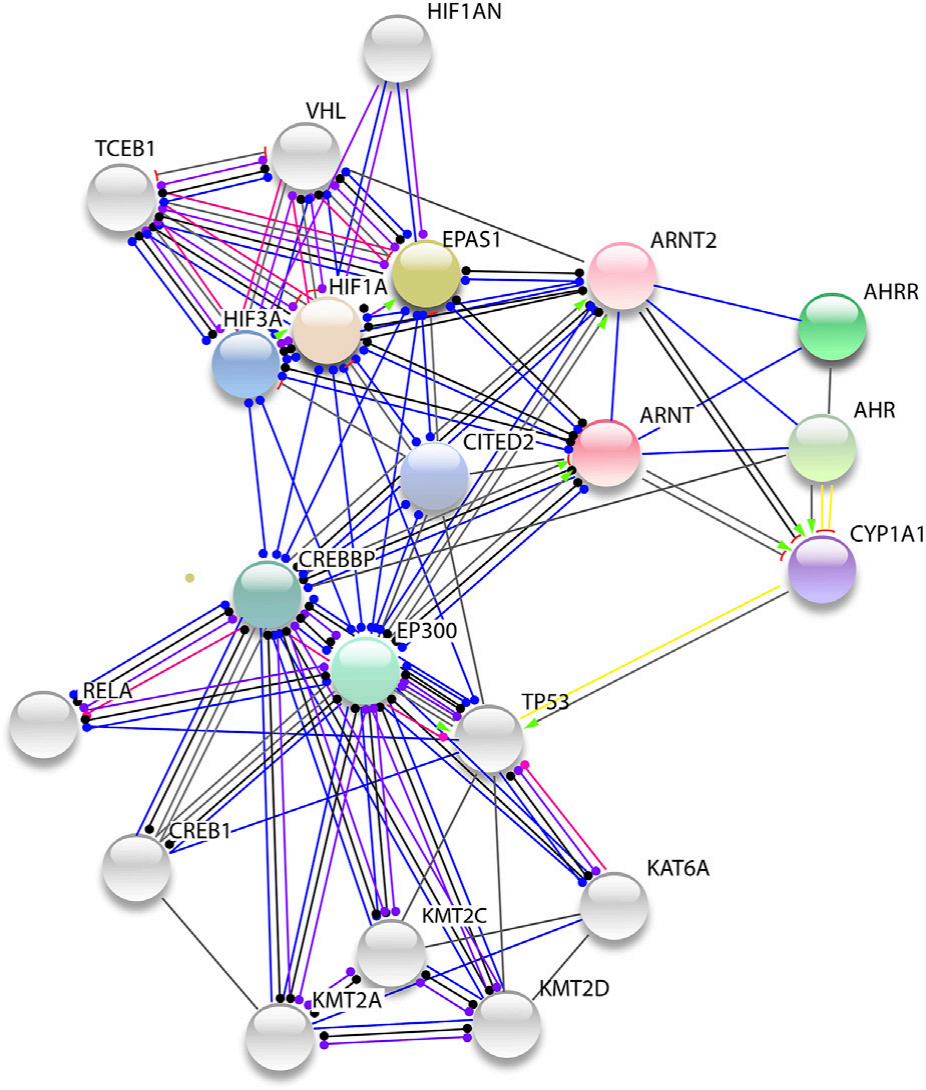
Protein-protein network of ARNT. The genes show several protein interaction networks interacting with hypoxia induced factors (HIF1a, EPAS1, CREBBP, and EP300).

*ARNT* also enhances endothelial cell growth in the vascular line and it is expressed during the early phase of the growth of new blood vessels (angiogenesis) (Scheinfeldt et al., 2012; Geng et al., 2014; Graham and Presnell, 2017). Protein-protein interaction network analysis shows that ARNT interacts with hypoxia-inducible factors such as HIF1a, EPAS1, EP300, and its paralog CREBBP (Figure 10). *CBFA2T3* is clustered in response to hypoxia and functions as a transcription regulator of *HIF1a* through interaction with *EGLN1* and promoting the *HIF1a* prolyl hydroxylation-dependent ubiquitination and proteasomal degradation pathways (Kumar et al., 2015). It also contributes to the inhibition of glycolysis and the stimulation of mitochondrial respiration by down-regulating the expression of glycolytic genes as direct targets of *HIF1a* (Kumar et al., 2013).

## Discussion

This study aimed to unravel at the autosomal genome level the adaptation of Ethiopian indigenous cattle to the extreme environmental conditions of its mountainous areas. We studied specifically three cattle populations of Semien, Bale, and Choke living in a mountainous area of more than 3,000 masl by contrasting them with the indigenous cattle population from the Ethiopian lowlands. Population genetic structure validated the African zebu admixture of indicine and taurine status of all the studied indigenous Ethiopian cattle (Figure 1), while the PCA result shows some level of genetic differentiation between the high-altitude Ethiopian cattle populations from those originating from the low altitude locations (Figure 2B). We then applied the iHS, XP-CLR, and PBS methods to detect selection signatures within and between the Ethiopian cattle populations living at high and low altitudes. Finally, additional comparisons of the candidate genomic regions between the high- and low-altitude cattle populations were carried out based on their nucleotide diversity, population differentiation, and haplotype LD heatmap differences for a detailed exploration of the high altitude adaptation.

### Novel candidate gene identified in this study

We identified three novel strong candidate genes (*CLCA2, SLC26A2,* and *CBFA2T3*) (Figure 6) for high altitude adaptation along with the previously reported *ITPR2* gene. The functional analysis clustered the candidate genes into ion channel activity (*CLCA2*, *SLC26A2*, and *ITPR2*), response to hypoxia (*ITPR2* and *CBFA2T3*), and renin secretion KEGG pathway (*CLCA2*) (Table 3). The renin pathway and ion channel activity regulate smooth muscle tone and epithelial secretion in response to hypoxia (Al-Hashem et al., 2012; Palubiski et al., 2020) by controlling arterial blood flow pressure (Shimoda and Polak, 2011). Hypoxia induces the expression of *CLCA2* in the pulmonary artery smooth muscle of rats and controls cell proliferation and apoptosis in the ERK1/2-MAPK signaling pathway (Huang et al., 2017; Zhao et al., 2017). Similarly, the renin secretion pathways maintain the amount of plasma renin and aldosterone concentration by modulating the normal relationship between plasma osmolality and plasma vasopressin concentration in humans (Bestle et al., 2002; Savoia et al., 2011). The renin-angiotensin and vasopressin function is stimulated by increased blood pressure caused by vesicular smooth muscle tone (Chassagne et al., 2000) to regulate high blood flow to balance cellular oxygen demand.

*SLC26A2* showed the highest PBS value in the high-altitude cattle populations (Figure 6). The haplotype LD heatmap, nucleotide diversity, and population differentiation index all supporting positive selection at the genome region overlapping with the gene (Figures 8, 9). The function of this gene is related to ion transport, and it plays a role in chondrocyte proliferation, differentiation, and growth in endochondral bone formation (Park et al., 2014). In humans, it regulates body size, and its recessive allele contributes to the dwarfism phenotype (Yang and Liang, 2021) and dysplasia (Pineda et al., 2013; Zheng et al., 2019; Heidari et al., 2021). A previous study reported a dominant allele at *SLC26A2* linked to higher heels and stronger claws in dairy cattle, while mutation at the gene causes dysplasia (Brenig et al., 2003). Considering the rugged and rocky terrain of the Ethiopian highlands, strong claws and high heels may prove advantageous. Further phenotypic characterization of the Ethiopian highland cattle may support this interpretation. The short stature and small body size of cattle observed in the Ethiopian high-altitude cattle confer the evidence. Though confirmatory analysis is required to differentiate the nature of short stature and small body size for HA adaptation in Ethiopian cattle, it could be a possible mechanism of the cold and high-altitude adaptation as it was reported in humans adapted to high altitude (Ma et al., 2019).

### Candidate convergent genome evolution between cattle and humans living in the Ethiopian highlands

The human population in Ethiopia occupied the high altitudes thousands of years ago, expanding from the lower Rift Valley in the early Pleistocene age (Aldenderfer, 2006). Archaeological evidence suggests that humans inhabited Bale Mountain approximately 50–30 thousand years ago (Ossendorf et al., 2019). Today, the human communities occupying the high altitude areas where the cattle samples were collected are the Oromo (Bale Mountain) and the Amhara (Semien and Choke mountains). The beginning of the settlement of the Amhara to these high-altitude regions is thought to have started around 5,000 years (Alkorta-Aranburu et al., 2012), while the settlement of the Oromo people was since early 1500s as reported by Hassen (1990) (cited in Alkorta-Aranburu et al., 2012; Huerta-Sánchez et al., 2013). The settlers in these territories were agrarian and had close interaction with their animals as sources of food and means of food production. Both humans and cattle living in the Ethiopian high altitudes share similar environmental challenges. Humans and ruminants living at high altitudes can be exposed to extended hypoxia stress and develop high-altitude sicknesses that may lead to high-altitude pulmonary hypertension (Friedrich and Wiener, 2020). For example, reports have indicated that cattle exposed to high altitudes may develop brisket disease caused by hypoxia (Newman et al., 2011; Wuletaw et al., 2011). Besides hypoxia, UV light and cold temperatures have been reported as major risk factors that challenge the survival of humans and other species in high altitude environments. Through a long-term evolutionary process, these risk factors may have induced positive selection pressures for physiological and morphological features that contribute to the evolutionary adaptation to high altitude environments (Witt and Huerta-Sánchez, 2019). Candidate genes detected in Ethiopian people living at high altitudes (Huerta-Sánchez et al., 2013), including *ITPR2*, *ARNT*, *EP300*, *MB*, and *HMOX1*, were also detected in Ethiopian cattle living in similar environments, supporting a convergent evolution between these two mammalian species.

Previous studies on the Ethiopian human population adapted to high altitudes have reported the *ITPR2* gene (Huerta-Sánchez et al., 2013) as a candidate gene. The *ITPR2* is also one of the candidate genes detected in HA cattle populations. It regulates vascular endothelial cells and intracellular calcium ion channel activity (Manalo et al., 2005; Jurkovicova et al., 2008). Following hypoxia, the cardiovascular system will increase blood flow by increasing pressure through vasoconstriction, increased heart rate, and myocardial contractility (Parati et al., 2015). These adaptive physiological mechanisms will enhance the supply of blood oxygen to tissues. *ITPR2* increases intracellular calcium concentration in vascular smooth muscle and it controls vasoconstriction avoiding pulmonary hypertension (Remillard and Yuan, 2006; Newman et al., 2011; Lai et al., 2015). *ITPR2*, as part of the calcium gated channel activities, also enhances endothelial cell proliferation lining and it triggers the vasculature and remodeling of the arterial tone to control the high blood pressure following hypoxic exposure (Makino et al., 2011; Hübner et al., 2015).

High altitude adaptation also depends on the concentration of hemoglobin in red blood cells and its affinity to oxygen in tissues (Alkorta-Aranburu et al., 2012). Also, increasing the number of erythrocytes will lead to higher hemoglobin concentration at the tissue level (Siebenmann et al., 2015). The candidate *MB* gene (Table 3) has been reported to play a role in increasing the hemoglobin concentration in muscle (Fraser et al., 2006; Jaspers et al., 2014) and increasing oxygen storage and binding affinity in hypoxic conditions (Hoppeler and Vogt, 2001; Li et al., 2018). This myoglobin gene was also reported under selection in the Ethiopian and Tibetan human populations living in highlands (Beall et al., 2002; Moore et al., 2002; Alkorta-Aranburu et al., 2012; Scheinfeldt et al., 2012). The gene is involved in erythrocyte homeostasis and regulates the level of hemoglobin (Avivi et al., 2010) in response to high altitude adaptation.

### Selection signatures overlap between Ethiopia cattle and other species adapted to high altitudes

Several studies have reported candidate positive selection signatures for high-altitude adaptation in different species. Here, besides the overlap with human candidate selected regions, we identified several candidate regions which aligned with genes reported under selection in other species adapted to high altitudes. They include *MSRB3* with the highest XP-CLR score in our study and *MC1R* previously reported under selection in Ethiopian highland sheep (Edea et al., 2019). The *MSRB3* gene was also reported in Tibetan dogs and sheep (Vaysse et al., 2011; Wei et al., 2016; Witt and Huerta-Sánchez, 2019). It has a pleiotropic effect in being involved in the ossification and adipose tissue development in cattle (Saatchi et al., 2014). It has also been linked to ear size in Tibetan sheep (Wei et al., 2016), pigs (Zhang et al., 2015), and dogs (Vaysse et al., 2011). The *MSRB3* gene also protects cells from oxidative stress caused in mammals by hypoxia (Hansel et al., 2005), while it is linked to cold and heat tolerance in Drosophila (Lim et al., 2012) and cold tolerance in Arabidopsis (Kwon et al., 2007). Cold temperature is one of the environmental stressors that trigger animal cells to transduce energy to adapt to cold temperatures.

Last but not least, among the genes present within candidate genomic regions detected by both XP-CLR and iHS analyses, we do have *ABCB10* (Table 3). This gene was previously reported in candidate selected regions in humans, and several other species, including cattle (Bayeva et al., 2013; Martinez et al., 2020). Its function is related to iron metabolism and heme biosynthesis (Haase, 2010; Shah et al., 2013; Yamamoto et al., 2014; Seguin and Ward, 2018). *ABCB10* is also involved in the transport of heme out of the mitochondria, before hemoglobinization of erythropoietic cells (Liesa et al., 2012; Bayeva et al., 2013).

## Conclusion

Despite the particularly challenging environmental conditions of the high-altitude Ethiopian highlands and the relatively recent arrival of African indicine cattle in these areas, we identified several genomic regions with evidence of positive selection for high-altitude environment adaptations at the autosomal level. These include genes previously reported in other mammalian species, including humans, living in high altitude areas in Ethiopia or other parts of the world, as well as in Ethiopian-specific cattle genomic regions. Our results show that these indigenous livestock populations are locally adapted, and they have developed a physiological mechanism to cope with the environmental challenges of hypoxia, UV radiation, and cold temperature. It calls for the conservation of these indigenous cattle adaptations as well as for their utilization in breeding programs combining the improvement of productivity with adaptability.

## Data Availability

The original contributions presented in the study are publicly available. The WGS data of Bale and Semien cattle samples are available at https://www.ncbi.nlm.nih.gov/bioproject/PRJNA698721, while the Choke cattle samples WGS data is available at https://www.ncbi.nlm.nih.gov/bioproject/PRJNA841948/.
